# Locked tight (mostly): Histone ubiquitylation by PRC1 isn’t always required for H3K27me3-dependent gene silencing

**DOI:** 10.1093/plcell/koaf291

**Published:** 2026-01-05

**Authors:** Rory Osborne

**Affiliations:** Assistant Features Editor, The Plant Cell, American Society of Plant Biologists; School of Biosciences, University of Birmingham, Birmingham B15 2TT, United Kingdom

Eukaryotic gene expression is regulated through the packing of genetic material into chromatin. Chromatin structure influences how effectively the transcriptional machinery can access coding regions of DNA, with its relaxation associated with active transcription and its compaction with gene repression. This highly dynamic process is regulated through histone modifications, which influence how tightly DNA associates with nucleosomes and how tightly nucleosomes associate with one another (Le et al. 2025). This additional layer of control affords complexity to multicellular life by regulating which genes are expressed, where they are expressed, and when they are expressed.

Many histone modifications have been described in both plants and animals. For example, trimethylation of lysine 27 on histone H3 (H3K27me3) is well understood to attenuate gene expression by promoting the formation of facultative heterochromatin. In plants, this modification is written by the Polycomb Repressive Complex 2 (PRC2) to establish a repressed state predominantly in developmental genes (Mozgova and Hennig 2015). PRC2 activity is further supported by PRC1, which operates in tandem by depositing histone H2A ubiquitylation (H2Aub) to maintain gene silencing (Zhou et al. 2017). Interestingly, H2Aub and H3K27me3 do not always colocalize within the genome, suggesting both PRC1 and PRC2 possess non-overlapping targets and functions (Tamburri et al. 2020).

To better understand this observation, **Minqi Yang and colleagues** (Yang et al. 2025) investigated the activity of 2 PRC1 E3 ligases: REALLY INTERESTING NEW GENE 1 A/B (RING1A/B) and B-cell-specific Moloney Murine leukemia virus integration site 1 A/B/C (BMI1A/B/C). The authors cloned wild-type and enzymatically dead variants of both proteins by mutating 4 conserved residues in their respective RING domains (RING1A4M, BMI1B4M) and used these constructs to complement *ring1ab* and *bmi1abc* knockouts in Arabidopsis. While global levels of H2Aub and H3K27me3 were restored when native RING1A or BMI1B were used to complement these mutants, both modifications remained depleted in RING1A4M and BMI1B4M. This was also reflected in the growth of respective genotypes, where the severe developmental phenotypes of *ring1ab* and *bmi1abc* were reverted by wild-type PRC1 components but not the enzymatically inactive ones.

After confirming that the interactions and localization of RING1A/RING1A4M and BMI1B/BMI1B4M had not been affected by these point mutants, the authors performed extensive chromatin immuno-precipitation sequencing to profile H2Aub and H3K27me3 levels in 10-day-old seedlings. The results revealed a stronger reduction in H2Aub in *ring1ab* than in that in *bmi1abc* for genes that were not marked by H3K27me3, suggesting that H2Aub on genes marked only by H2Aub mainly depends on RING1A/B. Both wild-type RING1A and BMI1B were able to restore H2Aub levels to those of *ring1b and bmi1ac*, respectively, while, as expected, H2Aub remained dramatically reduced in mutants complemented with the catalytically inactive variants. Interestingly, however, BMI1B4M increased H2Aub deposition at genes also targeted by RING1A, suggesting that BMI1B4M can support histone ubiquitylation at these sites in the presence of an active RING1A.

When considering H3K27me3 deposition in RING1A4M, the authors identified a cluster of genes with reduced H2Aub but partially restored levels of histone methylation. This interesting observation suggests that at specific loci, RING1A might recruit PRC2 independently of its E3 ligase activity to promote H3K27me3 deposition. Similarly, although to a far lesser extent, catalytically inactive BMI1B4M was also able to increase H3K27me3 levels across genes with unrestored levels of H2Aub (Fig.). Together, this work has advanced our understanding of the interactions between the histone modifications H2Aub and H3K27me3, and the requirement of the catalytic function of RING-domain proteins.

**Figure 1.**
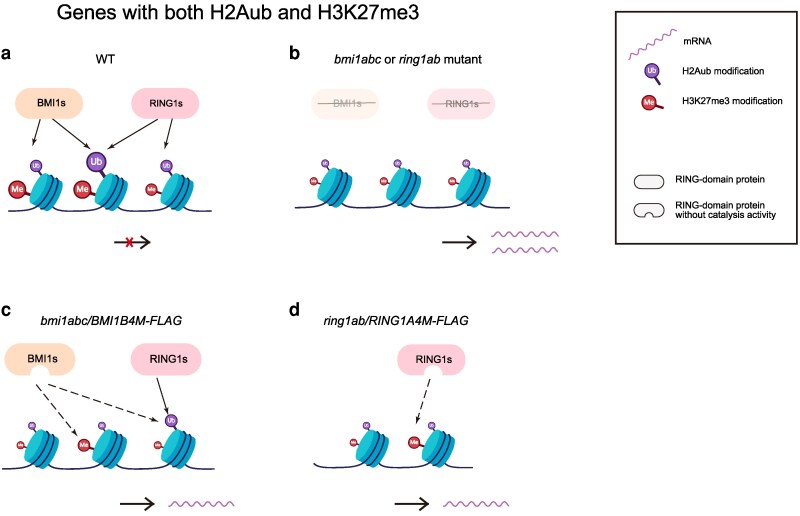
Model of BMI1 and RING1 activity in genes regulated by both H2Aub and H3K27me3 proposed by Yang et al. (2025). The level of modification is represented by the marker size (the 3 mutants represented on the right having lower levels than wild type on the far left). For genes possessing both H2Aub and H3K27me3, BMI1s and RING1s redundantly and uniquely regulate them to repress their expression (**a, b**). In the catalytically inactive mutants, the H3K27me3 levels of certain genes can be recovered (**c, d**). A catalytically inactive BMI1 can promote the function of RING1 to increase H2Aub levels on certain genes (c). Adapted from Yang et al. (2025), Figure 7.

## Recent related articles in *The Plant Cell*:

- Baile et al (2021) showed that transcription factor–mediated recruitment of PRC1/PRC2 to specific loci requires EAR-domain proteins to regulate gene expression and chromatin state dynamics.
- Yin et al. (2023) used Hi-C to map the chromatin architecture in both *PRC2* and *PRC1* mutant Arabidopsis, revealing the significance of both H3K27me3 and H2Aub in regulating chromatin loop formation and long-range DNA-DNA interactions.
- Samo et al. (2025) characterized the gene targets of PRC2 during seedling transition to photoautotrophy, showing that H3K27me3 is required to direct the metabolism away from seed storage molecules and toward products of photosynthesis.
